# Mutations of the transcription factor PU.1 are not associated with acute lymphoblastic leukaemia

**DOI:** 10.1038/sj.bjc.6603198

**Published:** 2006-05-30

**Authors:** B U Mueller, T Pabst, P Hauser, G Gilliland, D Neuberg, D G Tenen

**Affiliations:** 1 Department of Internal Medicine, University Hospital, Bern, Switzerland; 2 Department of Clinical Research, University Hospital, Bern, Switzerland; 3 Department of Medical Oncology, University Hospital, Bern, Switzerland; 4 Howard Hughes Medical Institute, Harvard Institutes of Medicine, Boston, MA, USA; 5 Department of Biostatistics, Dana-Farber Cancer Institute, Boston, MA, USA; 6 Harvard Institutes of Medicine, Harvard Medical School, Boston, MA, USA

**Keywords:** PU.1, ALL, transcription factor, mutation, leukaemia

## Abstract

The transcription factor PU.1 plays a crucial role during normal haematopoiesis in both myeloid cells and B-lymphocytes. Mice with a disruption in both alleles of the PU.1 locus were found to lack macrophages and B cells and had delayed appearance of neutrophils. In addition, critical decrease of PU.1 expression is sufficient to cause acute myeloid leukaemia (AML) and lymphomas in mice. Recently, we reported that heterozygous mutations in the PU.1 gene are present in some patients with AML. Thus, we hypothesised that PU.1 mutations might also contribute to the development of acute leukaemias of the B-cell lineage. Here, we screened 62 patients with B-cell acute lymphoblastic leukaemia (B-ALL) at diagnosis for genomic mutations by direct sequencing of all five exons of the PU.1 gene. We found no genomic alteration of the PU.1 gene suggesting that PU.1 mutations are not likely to be common in B-ALL.

PU.1, a member of the ets family of transcription factors, is upregulated during haematopoietic development, and is specifically expressed in mature myeloid and B cells (Klemsz *et al*, 1990; Hromas *et al*, 1993; Chen *et al*, 1995). The crucial and exclusive role of PU.1 in haematopoietic differentiation is established by the following observations. A number of genes are regulated by PU.1 in both myeloid and B-cell lineages, including those encoding colony-stimulating factor receptors and immunoglobulin subunits (Shin and Koshland, 1993; Zhang *et al*, 1994; Hohaus *et al*, 1995; Smith *et al*, 1996). Overexpression of PU.1 early in erythroid development blocks erythroblast differentiation and induces murine erythroleukaemia (Moreau-Gachelin *et al*, 1988, 1996; Paul *et al*, 1991). Gene targeting of PU.1 results in the failure of development of mature myeloid cells, and the addition of PU.1 site-containing competitor oligonucleotides to human CD34^+^ bone marrow cells inhibits *in vitro* myeloid colony formation (Voso *et al*, 1994; Fisher *et al*, 1998). In addition, mice with a disruption in both alleles of the PU.1 locus were found to have severe defects in the development of both myeloid and lymphoid cells, characterised by the absence of macrophages and B cells, and the delayed appearance of neutrophils (Scott *et al*, 1994; McKercher *et al*, 1996).

Although the role of PU.1 in normal haematopoiesis has been broadly investigated, its significance for leukaemogenesis is less known. Recently, it was shown that an 80% reduction of PU.1 expression is sufficient to cause acute myeloid leukaemia (AML) in mice (Rosenbauer *et al*, 2004). Our group has reported that heterozygous *PU.1* mutations are observed in some AML patients (Mueller *et al*, 2002). Finally, we demonstrated that PU.1 expression is critically suppressed by the PML-RARA fusion protein, the hallmark of AML-M3 patients (Mueller *et al*, 2006). These findings point to a role of *PU.1* for both normal haematopoietic differentiation and leukaemogenesis.

With regard to the finding of PU.1 mutations in some AML patients and to the role of *PU.1* in B-cell development, we hypothesised that B-cell acute lymphoblastic leukaemia (B-ALL) might similarly harbour genomic abnormalities in the *PU.1* gene. Therefore, we screened a collection of 62 B-ALL patients for *PU.1* mutations.

## PATIENTS AND METHODS

Analysis of mutations of the PU.1 gene was performed in 45 healthy volunteers (bone marrow donors) and 62 patients with B-ALL. Forty-six childhood B-ALL patients were treated on one of two consecutive Dana Farber Cancer Institute childhood ALL protocols (85-001 or 87-001), and 16 adult B-ALL patients were treated on the French Intergroup LALA 94 protocol in Bern, Switzerland. The 46 childhood ALL patients have previously been presented for unrelated purposes (McLean *et al*, 1996). Patient characteristics are presented in Table 1. The male/female ratio was 40/22 among the ALL patients and 29/16 for the healthy volunteers, and the age range was 1–62 years (median 9 years) for the ALL patients and 16–78 years (median 29 years) for the control group.

The patient samples were collected at the time of diagnosis before initiation of treatment and after having obtained informed consent. Mononuclear cells were isolated from bone marrow samples by Ficoll density gradient centrifugation and cryopreserved in liquid nitrogen until molecular analysis. Detection of PU.1 mutations was made on genomic DNA extracted from bone marrow samples. Polymerase chain reaction (PCR) analysis using exon-specific primers was performed of the five PU.1 exons comprising the entire coding region of the PU.1 gene. Exon-specific primers were designed from the sequence in GenBank (AC019059 and AC01841; Supplementary Table 2). All primers were selected to include the exon/intron boundaries to exclude splice junction mutations. Analysis of the PCR products was described previously (Mueller *et al*, 2002). Briefly, PCR products were electrophoresed on 1% agarose gels, gel-purified (Qiagen, Valentia, CA, USA), and sequenced using BigDye Terminators and AmpliTaq FS (Applied Biosystems, Foster City, CA, USA).

## RESULTS AND DISCUSSION

We screened leukaemic cells from patients with B-cell ALL for mutations in the coding region of the PU.1 gene by direct sequencing of exon-specific PCR products. We observed no sequence variants in the coding sequence in 62 ALL patients (90% upper confidence bound of 3.64%) and in 45 healthy volunteers (90% upper confidence bound of 4.98%).

The absence of detectable PU.1 mutations in ALL patients is in contrast to the results of our previous report where we detected mutations in a large series of AML patients (Mueller *et al*, 2002). In nine of 126 AML patients (7%), mutations of the PU.1 gene were identified, with seven of these nine AML patients having heterozygous mutations and two having both alleles affected by different mutations. Seven of the PU.1 mutations in AML patients affected the DNA-binding domain, and we demonstrated that those PU.1 mutations were deficient in DNA binding to and transactivation of the macrophage colony-stimulating factor receptor promoter, a direct PU.1 target gene. In addition, these mutations decreased the ability of PU.1 to synergise with interacting proteins such as AML1 or c-Jun to activate PU.1 target genes. Finally, some of these mutations have lost the ability of the PU.1 wild-type protein to induce terminal differentiation of PU.1-deficient progenitor cells.

Our results suggest that, unlike in AML, PU.1 is not inactivated by mutations in patients with ALL. Assuming a frequency of mutational events similar to that seen in AML patients (7%), we would expect to detect 4.34 mutations among the 62 ALL patients screened. Because the number of patients of each ALL subtype in our series is limited, we cannot exclude a low incidence of mutations in some specific subtypes of ALL. In addition, because we used exon-specific primers and DNA as a template, we cannot rule out the presence of some mutations not detectable by this PCR approach, for example, heterozygous deletions spanning one or several exons.

The apparent absence of PU.1 mutations in ALL in contrast to the presence of PU.1 mutations in some AML patients could be linked with the function of the PU.1 protein. It was recently hypothesised that differing concentrations of the PU.1 protein regulate the development of B-lymphocytes as compared with macrophages (DeKoter and Singh, 2000): A low concentration of PU.1 protein (one-fifth to one-seventh as much) was proposed to induce a B-cell fate, whereas a high concentration would promote macrophage differentiation and block B-cell development. It is noteworthy that wild-type macrophages express higher levels of PU.1 protein than their pro-B counterparts (Chen *et al*, 1995). This suggests that absence or reduction in the amount of wild-type PU.1 protein might have different consequences for myeloid and lymphoid development. Whereas PU.1 mutations might contribute to the malignant phenotype in myeloid cells, a mutant PU.1 peptide in lymphoid cells might lack consequences in terms of leukaemogenesis.

## Figures and Tables

**Table 1:** Clinical characteristics of patients studied

	**(*n*)**
*Protocol*	
85-001	22
87-001	24
LALA 94	16
	
*Age*	
Range (years)	1–62
Younger than 20 years	46
20 years or older	16
	
*Immunophenotype*	
B-cell	62
T-cell	0
	
*CNS involvement*	
No	57
Yes	5
	
*Sex*	
Male	40
Female	22
	
*WBC*	
<20 000/*μ*l	27
>20 000/*μ*l	35
	
*Cytogenetics*	
t(12;21)	11
t(9;22)	5
t(4;11)	2
t(1;19)	1
Complex	11
Normal	15
Others	13
NA	4

CNS=central nervous system; NA=not available; WBC=white blood cell count.

Clinical characteristics at the time of diagnosis of the 62 ALL patients are shown. Patients were enrolled on one of two consecutive Dana Farber Cancer Institute childhood ALL protocols (85-001 and 87-001) or on the French Intergroup LALA 94 protocol for adult ALL patients.

## References

[bib1] Chen HM, Zhang P, Voso MT, Hohaus S, Gonzalez DA, Glass CK, Zhang DE, Tenen DG (1995) Neutrophils and monocytes express high levels of PU.1 (Spi-1) but not Spi-B. Blood 85: 2918–2928 7742552

[bib2] DeKoter RP, Singh H (2000) Regulation of B lymphocyte and macrophage development by graded expression of PU.1. Science 288: 1439–1441 10827957 10.1126/science.288.5470.1439

[bib3] Fisher RC, Olson MC, Pongubala JM, Perkel JM, Atchinson ML, Scott EW, Simon MC (1998) Normal myeloid development requires both the glutamine-rich transactivation domain and the PEST region of transcription factor PU.1 but not the potent acidic transactivation domain. Mol Cell Biol 18: 4347–4357 9632818 10.1128/mcb.18.7.4347 PMC109018

[bib4] Hohaus S, Petrovick MS, Voso MT, Sun Z, Zhang DE, Tenen DG (1995) PU.1 (Spi-1) and C/EBP*α* regulate the expression of the granulocyte–macrophage colony-stimuating factor receptor *α* gene. Mol Cell Biol 15: 5830–5845 7565736 10.1128/mcb.15.10.5830 PMC230835

[bib5] Hromas R, Orazi A, Neiman RS, Maki R, Van Beveran C, Moore J, Klemsz M (1993) Hematopoietic lineage-restricted and stage-restricted expression of the ETS oncogene family member PU.1. Blood 82: 2998–3004 8219191

[bib6] Klemsz MJ, McKercher SR, Celada A, Van Beveren C, Maki RA (1990) The macrophage and B cell-specific transcription factor PU.1 is related to the ets oncogene. Cell 61: 113–124 2180582 10.1016/0092-8674(90)90219-5

[bib7] McKercher SR, Torbett BE, Anderson KL, Henkel GW, Vestal DJ, Baribault H, Klemsz M, Feeney AJ, Wu GE, Paige CJ, Maki RA (1996) Targeted disruption of the PU.1 gene results in multiple hematopoietic abnormalities. EMBO J 15: 5647–5658 8896458 PMC452309

[bib8] McLean TW, Ringold S, Neuberg D, Stegmaier K, Tantravahi R, Ritz J, Koeffler HP, Takeuchi S, Janssen JW, Seriu T, Bartram CR, Sallan SE, Gilliland DG, Golub TR (1996) TEL/AML1 dimerizes and is associated with a favorable outcome in childhood acute lymphoblastic leukemia. Blood 86: 4252–4258 8943861

[bib9] Moreau-Gachelin F, Tavitian A, Tambourin P (1988) Spi-1 is a putative oncogene in virally induced murine erythroleukaemias. Nature 331: 277–280 2827041 10.1038/331277a0

[bib10] Moreau-Gachelin F, Wendling F, Molina T, Denis N, Titeux M, Grimber G, Briand P, Vainchenker W, Tavitian A (1996) Spi-1/PU.1 transgenic mice develop multistep erythroleukemias. Mol Cell Biol 16: 2453–2463 8628313 10.1128/mcb.16.5.2453 PMC231234

[bib11] Mueller BU, Pabst T, Fos J, Petkovic V, Fey MF, Asou N, Buergi U, Tenen DG (2006) ATRA resolves the differentiation block in t(15;17) acute myeloid leukemia by restoring PU.1 expression. Blood 107: 3330–3338 16352814 10.1182/blood-2005-07-3068 PMC1895760

[bib12] Mueller BU, Pabst T, Osato M, Asou N, Johansen LM, Minden MD, Behre G, Hiddemann W, Ito Y, Tenen DG (2002) Heterozygous PU.1 mutations are associated with acute myeloid leukemia. Blood 100: 998–1007 12130514 10.1182/blood.v100.3.998

[bib13] Paul R, Schuetze S, Kozak SL, Kozak CA, Kabat D (1991) The Sfpi-1 proviral integration site of Friend erythroleukemia encodes the ets-related transcription factor PU.1. J Virol 65: 464–467 1985210 10.1128/jvi.65.1.464-467.1991 PMC240539

[bib14] Rosenbauer F, Wagner K, Kutok JL, Iwasaki H, Le Beau MM, Okuno Y, Akashi K, Fiering S, Tenen DG (2004) Acute myeloid leukemia induced by graded reduction of a lineage-specific transcription factor, PU.1. Nat Genet 36: 624–630 15146183 10.1038/ng1361

[bib15] Scott EW, Simon MC, Anastai J, Singh H (1994) The transcription factor PU.1 is required for the development of multiple hematopoietic lineages. Science 265: 1573–1577 8079170 10.1126/science.8079170

[bib16] Shin MK, Koshland ME (1993) Ets-related protein PU.1 regulates expression of the immunoglobulin J-chain gene through a novel Ets-binding element. Genes Dev 7: 2006–2015 8406004 10.1101/gad.7.10.2006

[bib17] Smith LT, Hohaus S, Gonzalez DA, Dziennis SE, Tenen DG (1996) PU.1 (Spi-1) and C/EBP*α* regulate the granulocyte colony-stimulating factor receptor promoter in myeloid cells. Blood 88: 1234–1247 8695841

[bib18] Voso MT, Burn TC, Wulf G, Lim B, Leone G, Tenen DG (1994) Inhibition of hematopoiesis by competitive binding of the transcription factor PU.1. Proc Natl Acad Sci USA 91: 7932–7936 7520173 10.1073/pnas.91.17.7932 PMC44518

[bib19] Zhang DE, Hetherington CJ, Chen HM, Tenen DG (1994) The macrophage transcription factor PU.1 directs tissue specific expression of the macrophage colony stimulating factor receptor. Mol Cell Biol 14: 373–381 8264604 10.1128/mcb.14.1.373-381.1994 PMC358386

