# Mutations of the transcription factor PU.1 are not associated with acute lymphoblastic leukaemia

**DOI:** 10.1038/sj.bjc.6603264

**Published:** 2006-08-15

**Authors:** B U Mueller, T Pabst, P Hauser, G Gilliland, D Neuberg, D G Tenen

**Correction to**: *British Journal of Cancer* (2006) **94**, 1918–1920. doi:10.1038/sj.bjc.6603198

Owing to an author error, G Gilliland was incorrectly named as contributing to this work. The authors would like it to be known that the correct list of authors who contributed was, therefore:

BU Mueller, T Pabst, P Hauser, D Neuberg and DG Tenen.

