# Supplementary material for: Mutations of the transcription factor PU.1 are not associated with acute lymphoblastic leukaemia
Source: Br J Cancer. 2006 May 30;94(12):1918–20. doi: 10.1038/sj.bjc.6603198 (PMC2361337; doi:10.1038/sj.bjc.6603198)
Supplement: Supplementary Table 2 [file 94-6603198x1.doc]

**Supplemental Table 2: Primer sequences for amplification of the PU.1 gene.**

| Primers used: |  |  |
| --- | --- | --- |
| 5'-TCACCCAGGGCTCCTGTAGCTCA-3' | sense | exon 1 |
| 5'-TCGTGGGCAGGCAGGCAGGCGTCC-3' | antisense | exon 1 |
| 5'-TGATGGGGACCAGCGTGCGGGGT-3' | sense | exon 2 |
| 5'-TCTCTCCAGACCCCAGGACCAGGC-3' | atisense | exon 2 |
| 5'-ACTATAACCTTTTCCTGCCCTGCC-3' | sense | exon 3 |
| 5'-AGCCTGTGTCAGCTTCCTGTGAAG-3' | antisense | exon 3 |
| 5'-TGCACTCCTTCTCTCCCCAGCTGACC-3' | sense | exon 4 |
| 5'-ACACACACGCGACTCGGTGGCGTG-3' | antisense | exon 4 |
| 5'-CCGGGCCCCTGTGCGTACGCAAGG-3' | sense | exon 5 |
| 5'-CCGGGAGCGTCCTCCCTGTGTCCG-3' | antisense | exon 5 |

Depicted are the exon-specific sequences of the primers used to amplify the PU.1 gene (GenBank accession numbers AC019059 and AC018410).
